# Intergenerational associations of adverse birth outcomes: A surveillance report

**DOI:** 10.1016/j.pmedr.2020.101226

**Published:** 2020-10-19

**Authors:** Dong Liu, Ge Lin, Dejun Su, James M. Alexender, Xiaoting Sun, Ming Qu

**Affiliations:** a University of Nebraska Medical Center, College of Public Health, 984355 Medical Center, Omaha, NE 68198-4355, United States; b Nebraska Department of Health and Human Services, 301 Centennial Mall South, Lincoln, NE 68509, United States; c University of Nevada, Las Vegas, 4505 S. Maryland Pkwy, Las Vegas, NV 89154, United States; d Tenth People’s Hospital Affiliated to Tongji University School of Medicine, 301 Yanchang Middle Rd, Zha Bei Qu, Shanghai 200072, China

**Keywords:** Low birthweight, Preterm birth, Birth history, Intergenerational associations, Recurrence, Siblings, Vital statistics

## Abstract

Nebraska births between 1995 and 2005 were followed until 2018 to look for intergenerational associations of low birth weight (LBW) and preterm birth (PTB). Results from generalized estimating equations revealed that mothers born LBW preterm were more likely to deliver LBW (adjusted OR 1.94, 95% CI 1.39–2.71) or preterm (adjusted OR 1.65, 95% CI 1.20–2.27) than mothers born with normal weight or at term. In addition, mothers who had an LBW sibling were 44% more likely to have an LBW infant (OR 1.44, 95% CI 1.04–2.00). A consistent finding was also observed for mothers who had a PTB sibling (OR 1.47, 95% CI 1.10–1.95). Mothers who were LBW at birth or had any LBW siblings, especially two or more siblings, were more likely to repeat this adverse birth outcome. The same association was also observed in mothers who were born preterm.

## Highlights

• A statewide assessment of intergenerational adverse birth outcomes in terms of preterm birth and low birth weight.
• Associating both mother and her siblings' birth records to the adverse birth outcomes.
• Found significant intergenerational associations of adverse birth outcomes.

## 1 Introduction:

Low birth weight (LBW) and preterm birth (PTB) are two common adverse birth outcomes. In 2018, 8.3% of newborns in the United States were LBW, and 11.7% were PTB (Department of Health and Human Services, 2019), costing billions of dollars in annual economic losses and long-term health deficits (Butler and Behrman, 2007, Wadhwa et al., 2009). A recent study based on the Nebraska state birth certificate registry found high likelihoods of reoccurrences of LBW or PTB among mothers with multiple singleton-births, which suggests the existence of a genetic predisposition (Su et al., 2018). Despite the potential for intergenerational studies (Klebanoff et al., 1984, Klebanoff and Yip, 1987, Smid et al., 2017) to provide more evidence to support a generational effect, they are difficult to perform (Qian et al., 2017). The current study used the same analytical framework and data source as Su et al. (2018) to investigate the relationships between the newborns and their mothers' adverse birth outcomes Table 1.

**Table 1:** A description of all the variables used in the analysis: Nebraska 1995–2018.

Variables	N	Percent
Total eligible pairs	5,118	
Birth Outcomes		
LBW (G3)	385	7.5%
PTB (G3)	456	8.9%
Maternal (G2) characteristics		
Formerly LBW	269	5.3%
Formerly PTB	288	5.6%
Had LBW sibling	371	7.2%
Had PTB sibling	527	10.3%
Number of LBW sibling		
0	4,747	92.8%
1	297	5.8%
2+	74	1.4%
Number of PTB sibling		
0	4,591	89.7%
1	413	8.1%
2+	114	2.2%
Age when delivery		
< 19	2,122	41.5%
Married	257	5.0%
Education		
< 12 years	1,594	31.1%
12 years (High School Degree)	2,024	39.5%
>12 years	1,500	29.3%
Had gestational diabetes	208	4.1%
Hypertension during pregnancy	377	7.4%
Tobacco used during pregnancy	830	16.2%
C-section	996	19.5%
Grandmother (G1) characteristics		
Age when delivery		
< 19	574	11.2%
19 – 34	4,199	81.9%
35 or older	345	6.7%
Married	2,710	53.0%
Education		
< 12 years	1,586	31.0%
12 years (High School Degree)	2,113	41.3%
>12 years	1,419	27.7%
Lived in rural areas	2,709	52.9%
Race/ethnicity		
Non-Hispanic White	3,608	70.5%
Hispanic	759	14.8%
Non-Hispanic Black	509	9.9%
Non-Hispanic Other	240	4.7%
Had gestational diabetes	132	2.6%
Hypertension during pregnancy	165	3.2%
Tobacco used during pregnancy	1,580	30.9%
C-section	851	16.6%

Note: The percentages in this table usually do not add up to 100% since there are missing values.

## 2 Methods

### 2.1 Data and procedure

Data were from the Nebraska Mother Index (NMI), a population-based birth registry providing birth certificate data linkages to matrilineal multiple births and intergeneration-matrilineal births. In the United States, state-level intergenerational data linkage studies were all based on specific projects, such as linking: 1). mothers born in Tennessee in 1959–1966 and their children in 1979–1984 (Klebanoff and Yip, 1987), 2). mothers born in 1956 to 1976 and their infants in 1989–1991 in Illinois (Coutinho et al., 1997, David et al., 2010, Collins et al., 2011), and 3). neonatal admissions during 1987–1995 in Washington State to maternal birth certificates (Emanuel et al. 1999). Besides confirming findings Tennessee and Illinois, studies in Washington State found maternal birthweight was also associated with specific clinical conditions, such as cesarean delivery, the risk of gestational diabetes mellitus, respiratory distress (Li et al., 2003, Shy et al., 2000, Strandjord et al., 2000, Williams et al., 1999). Other intergenerational studies include those using Virginia data (Chapman and Gray, 2014, Smid et al., 2017), Georgia and South Carolina data for a broader range of outcomes (Adams et al., 1997, Feng et al., 2013, Williams et al., 2013, Kramer et al., 2014, Fleischer et al., 2019). All those projected-based intergenerational studies were confined to specific study periods. The Nebraska Mother Index (NMI) is unique because it is a part of the state public health data infrastructure and an ongoing data linkage operation of the state digital birth certificate registry rather than relating to any research project (Lin and Qu, 2016).

We selected the first (G1, grandmothers or mother's mother), the second (G2, mothers), and the third (G3, newborns) generation from NMI, which contains information of every newborn's birth weight, gestational age (week), delivery pattern, mother's information, etc. This database indexed mothers through probabilistic linking mother's identifiable information, such as the mother's first name, last name, date of birth, social security number, and residential address (Lin and Qu, 2016). If a mother appears in the NMI, both her birth record and her newborn's record can be traced. The G2 mothers' sibling's information was generated from birth certificates related to G1 mothers. NMI for the current study is a secondary dataset that removed all the identifiable information.

All singleton births between 2010 and 2018 from the NMI as G3 (N = 247,007) and their mothers in G2 from 1995 to 2005 (total G2 = 112,327) were eligible for selection. According to Su et al. (2018), we also restricted G2 to singleton births. A birth weight equal or>500 g and gestational age between 22 weeks and 44 weeks were considered eligible. Maternal age at delivery<14 years old was excluded. The NMI uses G1 mothers as an index; all eligible G2 mothers' biological siblings' birth conditions were also linked to G3 infants. This process reduced eligible G2 and G3 to 108,067 and 237,731, respectively, which yielded 6,060 linked pairs. After further restriction on the first birth of each G2 mother, we obtained 5,118 pairs of G3 infants and their G2 mothers. This number is reasonable given that some mothers were from out of the states (not linkable), and some had not given birth yet.

### 2.2 Outcomes, indicators of heritable effects and covariates

In our study, LBW was defined as birth weights<2,500 g, and PTB was defined as having gestational ages at live delivery<37 weeks. Accordingly, four binary variables were created, indicating if a G3 infant was LBW or PTB and if the mother was LBW and PTB. The outcomes of interest were LBW and PTB of G3 infants. Six indicators of heritable effects included G2 LBW and PTB status, G2′s sibling LBW and PTB status, and the total number of LBW or PTB siblings (0, 1, or 2 + ) from each G2 mother. Mothers' demographic and medical history variables were included as covariates, including age at birth (<19 years, 19 – 34, or 35 + years), marital status (married or unmarried), educational level (<12 years, 12 years, or > 12 years), rurality (rural or urban), race/ethnicity (Hispanic, non-Hispanic White, non-Hispanic Black, or another non-Hispanic group), binary indicators of diabetes, hypertension, using tobacco during pregnancy, and C-section in the delivery. Rural or urban status, as well as race and ethnicity, were based on G2 at birth (Hispanic, non-Hispanic White, non-Hispanic Black, or another non-Hispanic group).

### 2.3 Statistical analysis

Following Su et al. (2018) to treat correlated G2 subjects from the same matrilineal families, we used a generalized estimating equation (GEE) or logistic regression for correlated data to generate both the adjusted and unadjusted odds ratios (ORs) and 95% confidence interval (CI) for LBW and PTB of G3 infants (Carey et al., 1993). Adjusted ORs were estimated by including all the significant control variables. Software SAS version 9.4 (SAS Institute Inc., 2017) was used for all statistical analyses. P-value < 0.05 was considered statistically significant in this study.

## 3 Results

Among 5,118 linked mothers, the LBW and PTB rates were 7.5% and 8.9%, respectively, formerly LBW and PTB mothers accounted for 5.3% and 5.6%, and 7.2% and 10.3% had at least one LBW or PTB siblings, respectively. They were dominated by non-Hispanic White (70.5%) and Hispanic (14.8%), with<9.9% non-Hispanic Black. Over 41% of G2 had teen pregnancies (<19 years old), only 5% of them were married, and over 70% only had a high school or less education. In addition, 4.1% had diabetes during pregnancies, 7.4% had hypertension, 16.2% used tobacco, and 19.5% received C-section.

In contrast, only 19% of maternal mothers (G1) were<19 years old when they delivered G2, and a majority of them (53%) were married. Over 30% used tobacco, and 16% received C-section.

In Table 2, the first four heritable effects all had greater percentages than their corresponding reference percentages. Both the adjusted and unadjusted odds ratios showed significant associations between G2′s and G3′s adverse birth conditions. Controlling for other variables, mothers who were born with LBW were 94% more likely (adjusted OR 1.94, 95% CI 1.39–2.71) to have LBW infants than mothers born with normal weight. Similar associations were found in PTB. Both LBW (adjusted OR 1.46, 95% CI 1.04–2.06) and PTB (adjusted OR 1.65, 95% CI 1.20–2.27) mothers were more likely to have PTB deliveries than the referent.

**Table 2:** Unadjusted and adjusted odds ratios of low birth weight or preterm birth according to maternal (G2) characteristics.

Birth history variables	LBW (G3)				PTB (G3)		
	%LBW	Unadjusted OR (95% CI)	Adjusted OR^a^ (95% CI)		%PTB	Unadjusted OR (95% CI)	Adjusted OR^a^ (95% CI)
Formerly LBW							
No	7.1%	1.00 (Ref)	1.00 (Ref)		8.6%	1.00 (Ref)	1.00 (Ref)
Yes	15.2%	2.15 (1.56, 2.98)***	1.94 (1.39, 2.71)***		13.8%	1.59 (1.14, 2.23)*	1.46 (1.04, 2.06)*
Formerly PTB							
No	7.3%	1.00 (Ref)	1.00 (Ref)		8.6%	1.00 (Ref)	1.00 (Ref)
Yes	10.8%	1.47 (1.02, 2.12)*	1.41 (0.97, 2.05)		14.9%	1.75 (1.28, 2.39)***	1.65 (1.20, 2.27)*
Had LBW sibling							
No	7.3%	1.00 (Ref)	1.00 (Ref)		8.8%	1.00 (Ref)	1.00 (Ref)
Yes	11.1%	1.52 (1.10, 2.11)*	1.44 (1.04, 2.00)*		10.0%	1.13 (0.81, 1.58)	1.14 (0.81, 1.61)
Had PTB sibling							
No	7.1%	1.00 (Ref)	1.00 (Ref)		8.5%	1.00 (Ref)	1.00 (Ref)
Yes	10.8%	1.51 (1.14, 2.01)*	1.47 (1.10, 1.95)*		12.5%	1.47 (1.14, 1.91)*	1.47 (1.13, 1.91)*
Number of LBW siblings^b^							
0	7.3%	1.00 (Ref)	1.00 (Ref)		8.8%	1.00 (Ref)	1.00 (Ref)
1	8.8%	1.21 (0.81, 1.80)	1.17 (0.78, 1.76)		8.4%	0.95 (0.64, 1.43)	0.99 (0.66, 1.49)
2+	20.3%	2.80 (1.67, 4.70)***	2.40 (1.42, 4.06)*		16.2%	1.84 (1.04, 3.27)*	1.71 (0.96, 3.06)
Number of PTB siblings^b^							
0	7.1%	1.00 (Ref)	1.00 (Ref)		8.5%	1.00 (Ref)	1.00 (Ref)
1	9.7%	1.35 (0.98, 1.88)	1.33 (0.95, 1.85)		11.6%	1.37 (1.01, 1.85)*	1.36 (1.01, 1.85)*
2+	14.9%	2.09 (1.28, 3.40)*	1.96 (1.20, 3.21)*		15.8%	1.86 (1.16, 2.99)*	1.85 (1.15, 2.99)*

a. All the maternal and grandmother's characteristic variables in the description were adjusted in the GEE model. The following variables had significant associations to the outcomes, including mother had gestational hypertension or gestational diabetes, mother used tobacco during pregnancy, mother received C-section, grandmother was African American, and grandmother lived in the urban area.

b. The number of maternal PTB/LBW siblings is among all G2 women regardless.

* 0.01 < P < 0.05. *** P < 0.001.

In addition, mothers who had any LBW siblings were 44% more likely (adjusted OR 1.44, 95% CI 1.04–2.00) to have LBW infants than the comparison group. The adjusted OR was strongest when maternal LBW siblings were equal or greater than two (adjusted OR 2.40, 95% CI 1.42–4.06). A consistent finding was, likewise, observed for mothers who had any PTB sibling (adjusted OR 1.47, 95% CI 1.10–1.95). Mothers who had one PTB sibling were significantly associated with delivering PTB infants (adjusted OR 1.36, 95% CI 1.01–1.85), and this association was stronger when they had two or more PTB siblings (adjusted OR 1.85, 95% CI 1.15–2.99).

## 4 Discussion

In this study, we examined intergenerational correlations of LBW and PTB focusing on the subsequent pregnancy and their siblings. The study is a direct extension of the longitudinal study of adverse birth outcomes of the mother of multiple births (Su et al., 2018) to their daughters and daughter's infants. We found that mothers who were born with LBW were 94% more likely to have LBW infants than mothers who had normal birth weights. In addition, mothers who were preterm at birth were 46% more likely to have PTB deliveries. These two findings suggest the existence of intergenerational correlations of adverse birth outcomes (LBW or PTB) with potential genetic predispositions. These results were broadly consistent with findings from two early statewide matrilineal studies of LBW (Klebanoff and Yip, 1987) and PTB (Smid et al., 2017).

The flexibility of NMI in indexing birth outcomes from G1 mothers, allowed us to examine a mother's odds of having adverse birth outcomes according to her siblings' LBW or PTB status. If a mother had two or more LBW or PTB siblings, she was at least 71% to 140% more likely to have an LBW or PTB baby, respectively. This suggests that a G2 mother did not necessarily have to be born with an adverse outcome; if two or more of her siblings were born with an adverse outcome, she would have a greater likelihood of delivering LBW or PTB offspring. These findings to our knowledge have not been reported in previous studies in the United States.

Heritable effects, such as strong matrilineal relationships of LBW and PTB, seen in this study, can be due to genetic predisposition but may also represent socioeconomic or behavioral traits that are passed down through the generations. The adjusted odds ratios in the current study were consistent with a previous study using the Illinois transgenerational dataset (Collins et al. 2011): controlling for neighborhood SES, former LBW or PTB mothers had 30% to 80% greater odds of delivering LBW or PTB infants regardless race. Moving from low-income to high-income neighborhoods or persistently staying in high-income neighborhoods between generations had comparable LBW or PTB rates among African-American mothers. It is difficult to distinguish further effects of genetic inheritance from both socioeconomic and behavioral factors. For example, the recurrence of intergenerational teenage pregnancies has been reported (Meade et al., 2008, Liu et al., 2018), but pregnancy can be thought of as outcome, and teenage pregnancy may be a risk factor for LBW and PTB, due to learned behavior. Future studies are needed to disentangle these effects, especially using more elaborated SES variables between generations (Fleischer et al., 2019).

This study has several limitations. First, since the starting time for G2 follow-up was January 1995, the G2 mothers in our sample well<24 years old. For this reason, our findings are biased toward young age mothers. This bias might contribute to non-significant results of the education and marital status variables in all the multivariate analyses. Second, the 1995 to 2004 Nebraska birth certificate data did not record mothers' height and weight, and we were unable to include this information for gestational weight gain as confounders (Goldstein et al., 2017). Third, most health behavior variables (e.g., smoking) were collected by nurses prior to the delivery, and they were subject to mothers' recall bias, and some may have underreported behaviors due to social stigma. Fourth, NMI may have some mismatches, but they are unlikely to affect the results substantially. Finally, we do not have the childhood socioeconomic status of mothers, which might have affected their offspring's birth weight (Gavin et al., 2011). Since many of those variables can be derived directly by linking state government data system (welfare and child development) and indirectly through geocoding for neighborhood variables (Kramer et al., 2014), future work can expand our current analyses through further data integration to NMI.

## 5 Conclusions

Our results showed a strong intergenerational correlation of LBW or PTB. Mothers who were LBW at their own birth or had any LBW (especially two or more) siblings were much more likely to repeat this adverse birth outcome than their corresponding comparison groups. The same pattern was observed for PTB.

## CRediT authorship contribution statement

**Dong Liu:** Conceptualization, Methodology, Software, Validation, Formal analysis, Investigation, Data curation, Writing - original draft, Supervision, Project administration. **Ge Lin:** Conceptualization, Methodology, Data curation, Investigation, Writing - review & editing. **Dejun Su:** Writing - review & editing. **James M. Alexender:** Writing - review & editing. **Xiaoting Sun:** Writing - review & editing. **Ming Qu:** Investigation, Resources, Writing - review & editing.
